# Crystal structures and low-affinity complex formation of halogenase CtcP and FAD reductase CtcQ from the chlortetracycline biosynthetic pathway

**DOI:** 10.1042/BSR20253185

**Published:** 2025-07-04

**Authors:** Caixia Hou, Sylvie Garneau-Tsodikova, Oleg V. Tsodikov

**Affiliations:** Department of Pharmaceutical Sciences, College of Pharmacy, University of Kentucky, 789 South Limestone Street, Lexington, KY 40536, U.S.A.

**Keywords:** biosynthesis, chlorination, flavin, natural product, two-component system, *Streptomyces aureofaciens*

## Abstract

Enzymatic halogenation in natural products has been intensely investigated due to its potential utility as a tool to improve pharmacological and pharmaceutical properties of drug leads. Chlortetracycline (CTC), the first tetracycline (TC) antibiotic discovered nearly eight decades ago, contains a chlorine group. This chlorine is installed enzymatically by the flavin adenine dinucleotide (FAD)-dependent halogenase CtcP. CtcP and the FAD reductase CtcQ, which is also encoded in the CTC biosynthetic gene cluster, function as a two-component system. Structural information on CtcP and CtcQ has been lacking. In this study, we determined crystal structures of CtcP from *Kitasatospora aureofaciens* in a complex with polyethylene glycol and sulfate ions and in a complex with FAD, and a crystal structure of CtcQ in a complex with FAD and NAD. The structures of CtcP revealed a close similarity of this enzyme to the phenolic halogenase PltM, despite a large difference in the sizes of their respective substrates, presumably TC and phloroglucinol. The CtcP structure showed a conserved dimeric organization also found in PltM crystals. We showed that dimerization of CtcP is allosterically influenced by a distant C-terminal helical hairpin. A closed substrate-binding cavity of CtcP suggested that conformational changes were required to allow a substrate, likely not TC, to bind CtcP. We demonstrated that CtcP and CtcQ weakly bound each other. The dimeric structures of CtcP and CtcQ prompted us to propose approximate models of a 2:2/CtcP:CtcQ complex, where FAD(H_2_) would shuttle between the two enzymes for chlorination and reduction.

## Introduction

Enzymatic halogenation is an activity that has been found in bacteria and fungi. Halogenated natural products isolated from higher organisms, such as worms and sponges, may, in fact, all originate from their commensal microbiomes. Over 5,000 natural products contain a halogen [1], and most of these molecules are chlorinated or brominated. Halogenation is a useful modification in medicinal chemistry, since halogens have been observed to enhance bioactivity, as well as pharmacological and pharmaceutical properties of compounds [2–5]. Given these beneficial characteristics, it is not surprising that approximately 25% of the drugs used in clinic contain halogens [6]. For the last two decades, enzymatic halogenation has been a subject of intense inquiry at a fundamental level and as a potential biocatalytic tool due to its regiospecificity and environment-friendly process chemistry, two desirable attributes commonly lacking in halogenation performed by organic chemical synthesis.

FAD-dependent halogenases are especially interesting in this regard because of their high degree of regiospecificity. Groundbreaking studies of tryptophan halogenases RebH [7] and PrnA [8] revealed that these enzymes shared the same fold and belonged to the same structural family as flavin-dependent monooxygenases. In the halogenases, the enzyme-FADH_2_ complex reacts with molecular oxygen to form a flavin C4a-peroxide adduct [9], which activates a halide ion into a reactive hypohalous acid HOX. HOX then travels in a short protein channel protected from other reactions to a conserved lysine residue. HOX was proposed either to react with the lysine to form a haloamine adduct or to form hydrogen bonds with this lysine and an adjacent glutamate residue prior to halogenating the substrate [8]. A recent study of tryptophan halogenases demonstrated that FAD-dependent halogenases leaked HOX unproductively, independent of whether the active site lysine was mutated or not, favoring the hydrogen bonding mechanism of HOX over the haloamine adduct formation and explaining a relatively low catalytic efficiency of these enzymes [10]. Mutagenesis studies of the substrate-binding site of tryptophan halogenase RebH led to a modified substrate selectivity [11]. Apparently, the substrate selectivity of tryptophan halogenases has been greatly diversified by evolution. One example is provided by an elegant study of fungal halogenase MalA, which halogenates a complex alkaloid, where an indole group is a part of a large ring system [12].

**Figure 1:**
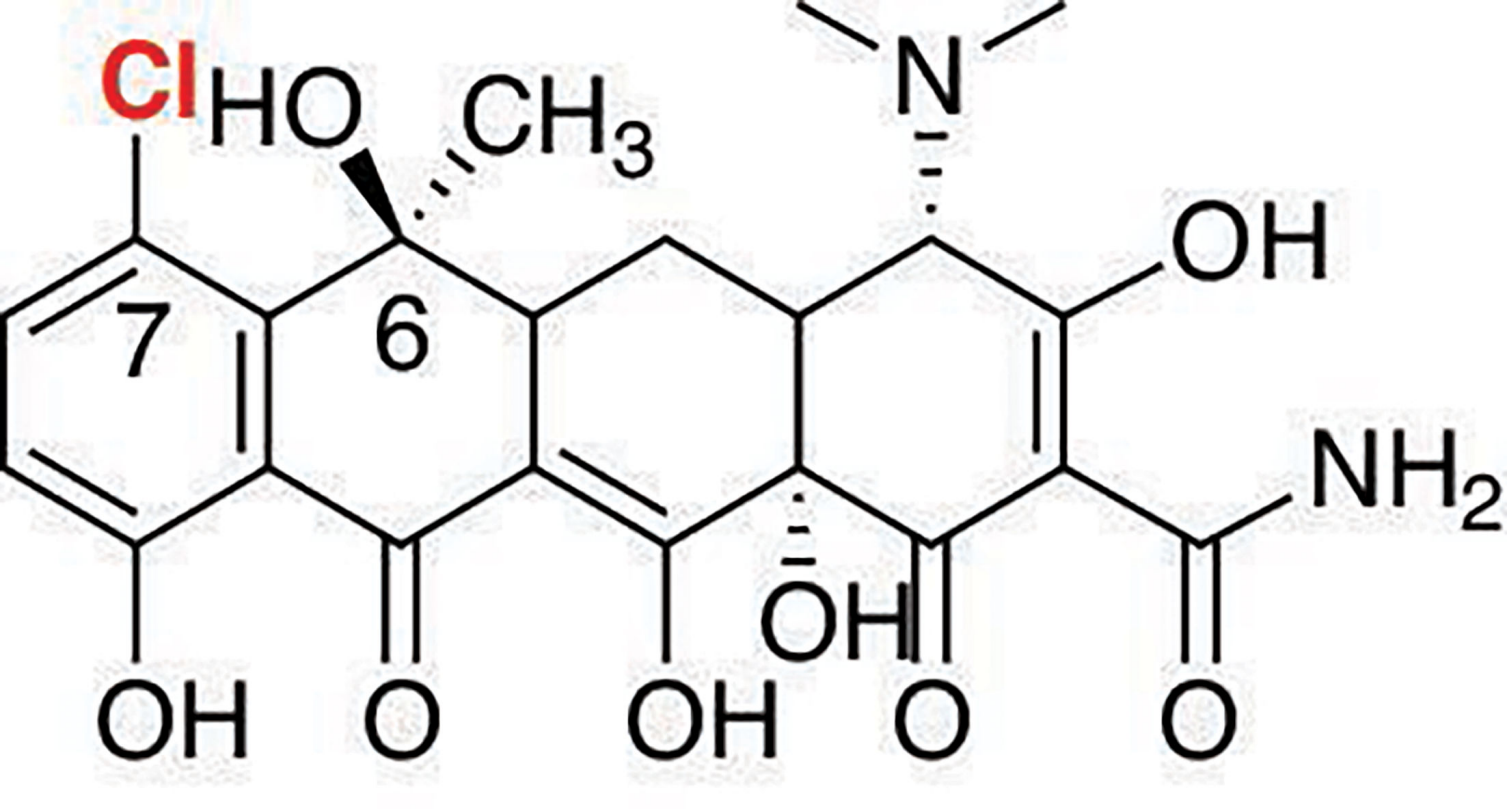
The structure of chlortetracycline (CTC).

While tryptophan halogenases have been most studied, much less is known about halogenases that modify other structures. Phenolic halogenases modify phenol and phenolic moieties incorporated in other structures, such as macrolactones [13]. We recently structurally and biochemically characterized a phenolic halogenase PltM from the pyoluteorin biosynthetic gene cluster of *Pseudomonas fluorescens* Pf-5 [14], after it was demonstrated that the endogenous substrate of PltM was phloroglucinol [15]. In addition to phloroglucinol, PltM chlorinated, brominated, and iodinated a variety of phenolic compounds, including resveratrol [14]. To understand the origins of substrate selectivity among phenolic halogenases, we aimed to investigate a phenolic halogenase that halogenated a phenolic substructure in the context of a larger molecule. Tetracycline (TC) halogenase CtcP from the bacterium *Kitasatospora aureofaciens* stood out, as it was reported to halogenate the C-7 position of TC to yield chlortetracycline (CTC) (Figure 1) [16]. These authors proposed that the halogenation catalyzed by CtcP was the last step of CTC biosynthesis in *K. aureofaciens*. This proposal was based on TC accumulation by the *ctcP* knockout strain. In the test tube, that study showed that TC was halogenated by purified CtcP, but the halogenation was inefficient and incomplete, even in the presence of an FAD regenerating system. Because TCs are clinically used antibiotics, understanding this biotransformation could have added practical value in the engineering of new TC analogs. CtcP is a part of a two-component system with FAD reductase CtcQ, an enzyme also encoded in the CTC gene cluster [16]. As with other halogenases and homologous oxygenases, their coupled FAD reductases regenerate FADH_2_. Interactions between the two enzymes of such two-component systems are usually weak, making them difficult to study. Because CtcQ and its interactions with CtcP have not been investigated previously, these interactions were also a subject of this study. When this study was ongoing, we became aware of a short report on the crystal structure of SeMet-substituted apo-CtcP in a different crystal form [17]. Our study only briefly reiterates general structural features of the enzyme described in that study while providing additional structural and functional information on both CtcP and CtcQ.

## Results and discussion

### A crystal structure of CtcP in complex with polyethylene glycol and sulfate

**Figure 2:**
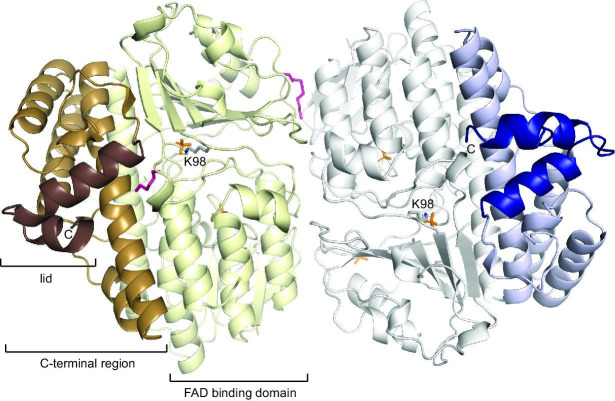
The crystal structure of the dimer of CtcP in complex with sulfate and PEG. The conserved FAD-binding domains (residues 1–406) in the two protomers are shown in pale yellow and white, the C-terminal regions structurally similar to those in PltM (residues 407–516) are in caramel and light blue, and the lid regions unique to CtcP (residues 517–555) are shown in brown and dark blue. The C-terminus is indicated by the letter C. The bound PEG fragment is shown by red sticks, the sulfate ions are shown by orange sticks. PEG, polyethylene glycol.

We cloned, overexpressed, purified by a combination of Ni^2+^-chelating and size-exclusion chromatography, and crystallized CtcP from a CTC producer *K. aureofaciens*. The crystal structure of CtcP (Figure 2) was determined by molecular replacement, using our crystal structure of phenolic halogenase PltM as a search model [14]. An asymmetric unit of the crystal contained one dimer of CtcP. Among the proteins that were structurally characterized, PltM was the most similar to CtcP, with a sequence identity of 37% over 491 out of 555 residues of CtcP. Other structurally characterized halogenases were much more dissimilar, with the closest among them being PltA (27% identity for 424 residues; Supplementary Figure S1), another FAD-dependent halogenase from the pyoluteorin biosynthetic gene cluster, also characterized by our group [18]. The structure superimposition and sequence alignment of CtcP with PltM and PltA (Supplementary Figure S1) showed that the first ~400 residues were most conserved in all three proteins. This N-terminal region is the FAD-binding domain present in all FAD-dependent halogenases. As expected, the FAD-binding domain of CtcP contained a conserved catalytic lysine residue (Lys98; Figure 2 and Supplementary Figure S1) that in other halogenases was proposed to form a haloamine intermediate or a hydrogen bond with hypohalous acid immediately prior to the halogenation step [8]. Both CtcP protomers contained a bound sulfate ion that formed a salt bridge with Lys98. The remaining C-terminal helical region was not conserved in PltA or other halogenases, whereas the structural similarity between CtcP and PltM extended to residue 516 of CtcP. The extreme C-terminal region of CtcP (residues 517–555) that was unique to this protein formed a helical hairpin (Figure 2). The substrate-binding site in the phenolic FAD-dependent halogenases is located in the cleft between the FAD-binding domain and the C-terminal region [14,18]. In one of the two CtcP molecules in the asymmetric unit, the substrate-binding site in CtcP was occupied by a molecule that was consistent with an ordered fragment of polyethylene glycol (PEG) from the crystallization solution (Figure 2). The PEG was bound to the site analogous to the phloroglucinol-binding site in PltM [14]. The C-terminal helical hairpin appeared to cover the entrance to the substrate-binding cleft like a lid.

### Dimerization of CtcP and its allosteric link with the extreme C-terminal region (residues 517–555)

**Figure 3:**
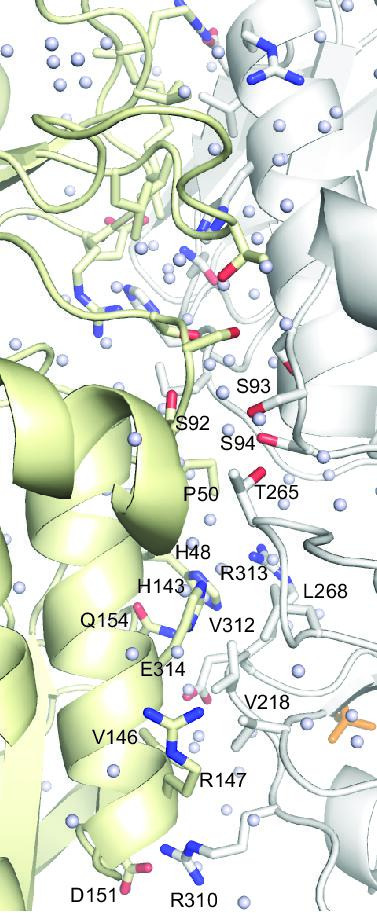
The dimeric interface of CtcP. The interface residues are shown as sticks, the interface water molecules are shown as light grey balls.

**Figure 4:**
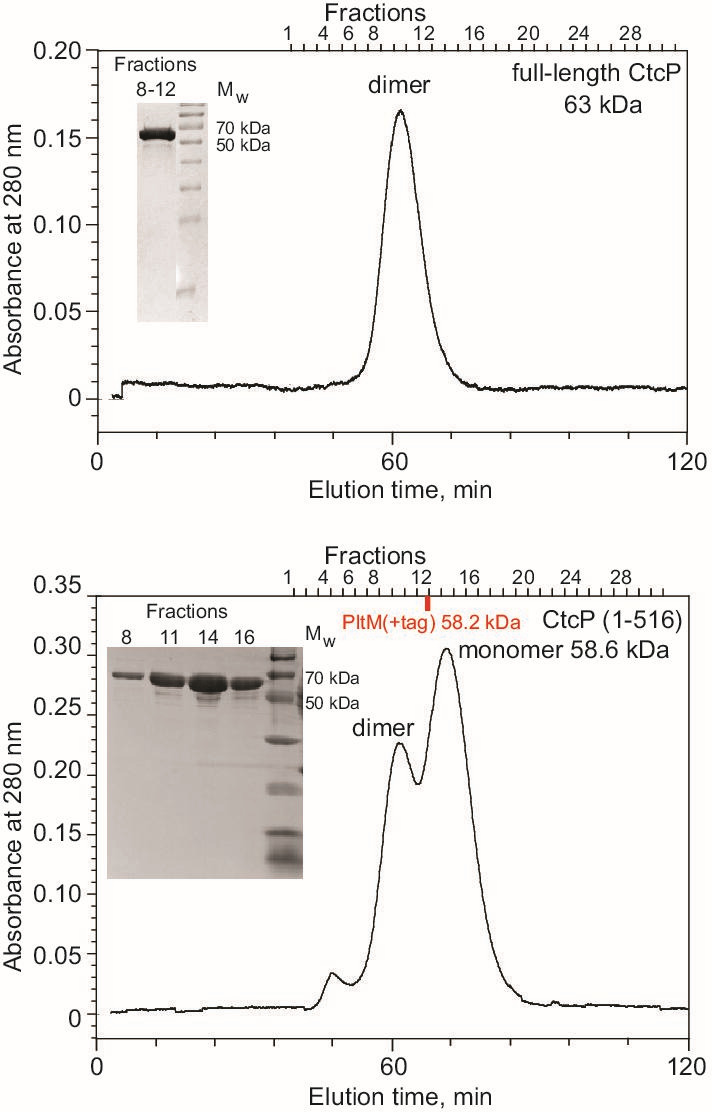
The size-exclusion chromatography of full-length CtcP (top) and its C-terminal truncation mutant CtcP (1-516) (bottom). The position of the monomeric PltM eluted using the same protocol is marked by a red tick. The tagged PltM has a very similar molecular weight as truncated CtcP (1-516), but the tag of PltM (10 kDa) makes this protein behave as a slightly larger protein on the size-exclusion column. The insets are images of 15% SDS-PAGE gels of the proteins from the specified fractions, demonstrating that all the relevant chromatographic peaks correspond to the expected proteins, and not their degradation products.

The dimer of CtcP is formed by the FAD-binding domains of two protomers related by two-fold rotational symmetry (Figures 2 and 3). The dimeric state of CtcP in the crystal is consistent with its dimerization in solution, as reported previously [16] and corroborated by our size-exclusion chromatography (Figure 4). The crystal structure of SeMet-containing apo-CtcP [17] contained two analogous dimers per asymmetric unit in a different crystal form, serving as an independent validation of the dimeric state, although a monomeric state was erroneously assigned to CtcP in that Protein Data Bank entry (PDB ID: 7XGB). The dimeric interface (Figure 3) buried 2,355 Å^2^ of solvent accessible surface area, which was 57% nonpolar and 43% polar. At the edge of this interface, Arg310 of one protomer formed a salt bridge with Asp151 of the other protomer. Hydrophobic interactions between the protomers included Val218 with an aliphatic stem of Arg147, as well as Val312 and L268 with the imidazole ring of His143. Several direct and numerous water-mediated hydrogen bonds also formed across the interface; these included a bond between the main chain carbonyl oxygen of Val312 and the side chain amide of Gln154, between a carboxyl oxygen of Glu314 and the main chain nitrogen of Gln154, and between the hydroxyl oxygen of Ser93 and the amide nitrogen of Ser93 of the other protomer. These and other residues involved are shown in Figure 3 and marked in Supplementary Figure S1. Analogous reciprocal interactions were formed in the other, symmetry-related, half of the interface. In examining our previously published crystal structure of PltM, we found that PltM formed similar dimers in the crystal, although 25% of the interface residues in PltM were not identical or homologous to those of CtcP. We showed that PltM was monomeric in solution [14]. We reasoned that the differences in the interface residues might contribute to the weakened propensity of PltM for dimerization in solution.

A previous study, an aim of which was to optimize CTC biosynthesis in a fermentation system, showed that the chlorination by CtcP was a slow step in the pathway and suggested that this was the last step in the CTC biosynthesis, converting TC to CTC [16]. We tested FAD-dependent chlorination of TC by full-length CtcP in the presence of FAD reductase CtcQ analogously to how we previously assayed the highly related PltM [14]. CtcP did not display TC chlorination activity observable by HPLC (Supplementary Figure S2). The above-mentioned study reported a weak catalytic efficiency of this reaction, which was significantly incomplete and measurable only when CtcP was both purified and assayed anaerobically [16]. To test whether the C-terminal helical harpin that appeared to block the access of the substrate to the active site was autoinhibitory, we generated and tested a mutant of CtcP (1-516) lacking this hairpin. As with the full-length CtcP, this deletion mutant did not appear to chlorinate TC. The lack of robust catalytic activity raised doubts that the chlorination of TC by CtcP was the last transformation in the CTC biosynthesis. In addition to the weak activity observed previously, the main argument for TC chlorination was previously made based on the accumulation of TC by the bacterial producer lacking the functional *ctcP* gene [16]. The weak *in vitro* catalytic activity of CtcP with TC as a substrate is highly suggestive of an alternative mechanism, where chlorination takes place earlier in the biosynthetic pathway, at least prior to 6-methylation by CtcK, while the subsequent biosynthetic enzymes accept either chlorinated or unchlorinated intermediates, explaining TC formation without functional CtcP. Another piece of strong and direct evidence in favor of this proposal is the formation of demeclocycline, the TC lacking the 6-methyl group as well as the chlorine installed by CtcP [19]. Therefore, we suggest that a preferred CtcP substrate in the CTC biosynthetic pathway is yet to be established.

While purifying the CtcP (1-516) lacking the C-terminal hairpin, we were surprised to find out that this deletion mutant eluted as a mixture of dimers and monomers on a size-exclusion column, with the monomers being the dominant species (Figure 4). The C-terminal helical hairpin is located far from the dimerization interface (Figure 2); therefore, the effect of this deletion on the dimerization equilibrium is allosteric, propagating from the C-terminus through the catalytic domain. The lack of the C-terminal helical hairpin in PltM is consistent with its monomeric state in solution.

### Substrate and FAD-binding sites of CtcP

**Figure 5:**
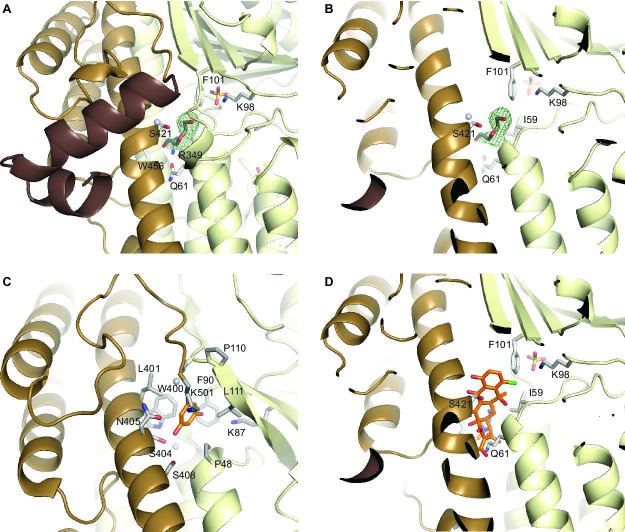
The substrate-binding pockets of CtcP and PltM. (**A**) The substrate-binding pocket of CtcP. The mesh is the *F*_o_-*F*_c_ omit map contoured at 3σ indicative of a bound PEG fragment (grey sticks). Also in sticks are residues lining the pocket and a sulfate ion. (**B**) A cut-away view of the substrate-binding pocket of CtcP. (**C**) The substrate-binding pocket of PltM from our previously published crystal structure (PDB ID: 6BZA). The bound phloroglucinol is shown by orange sticks. (**D**) The docking model of TC (orange sticks) in the substrate-binding pocket of CtcP. The TC sterically clashes with CtcP. PEG, polyethylene glycol.

The substrate-binding site of CtcP is unambiguously located based on its structural homology with PltM (Figure 5A–C) and on the location of the conserved catalytic Lys residue (Lys98 in CtcP and Lys87 in PltM). A strong difference in electron density in the substrate-binding site of CtcP was consistent only with an ordered portion of a PEG molecule from the crystallization solution (Figure 5A and B). The PEG was bound at a location analogous to that of the endogenous substrate of PltM, phloroglucinol (PDB ID: 6BZA; Figure 5C). A sulfate ion bound in the vicinity of the PEG formed a salt bridge with Lys98, likely mimicking the reactive hypochlorite. As in PltM, the substrate-binding site in CtcP is located in a cleft between the catalytic domain and the C-terminal region. The residues lining the substrate-binding site in CtcP are different from those in PltM, underscoring the very different substrates of the two enzymes. These residues in CtcP are polar (Gln61, Ser421), charged (Arg349), and hydrophobic (Ile59, Phe101, Trp456). Remarkably, the substrate-binding site of CtcP is rather small and similar to that of PltM, despite the considerable difference between a putative tetracyclic substrate of CtcP and a much smaller phloroglucinol. In fact, when modeling a TC molecule in the binding site, steric clashes with the side chains and the backbone could not be avoided (Figure 5D). One has to conclude that significant global conformational changes occur to accommodate a tetracyclic molecule in the substrate-binding cleft or that the chlorination substrate is an early intermediate in the pathway that is either not highly elaborated or even fully cyclized. Analogously, we previously concluded that large conformational changes must occur in the structurally related halogenase PltA, to allow its large substrate, a peptidyl carrier protein conjugate (PltL-pyrrolyl), to access the active site [18]. As we noted above, an earlier intermediate in the pathway that could fit the substrate-binding cleft would also be logical.

**Figure 6:**
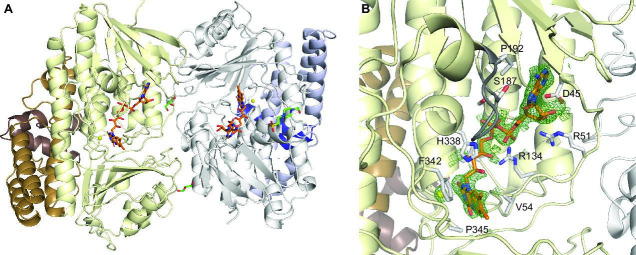
The crystal structure of CtcP in complex with FAD and Cl^-^. (**A**) The overall view of the CtcP dimer-FAD-Cl^-^ complex. The bound FAD is shown by orange sticks. (**B**) A zoomed in view of the FAD-binding cleft. The mesh is the *F*_o_-*F*_c_ omit map contoured at 3σ. The FAD interacting residues are shown by white sticks. The FAD sensor loop (residues 186–192) is colored dark grey.

The purified CtcP was not bound to FAD, indicating that FAD was not stably bound to the enzyme, a property that was also observed for PltM. This mechanism is likely used to allow a partner FAD reductase to bind and reduce the FAD for its reuse in halogenation. We obtained crystals of the CtcP-FAD complex by soaking FAD into the CtcP crystals and determined the crystal structure of the complex (Figure 6A). A molecule of FAD and a catalytic chloride ion bound at 3.3 Å from the isoalloxazine ring of the FAD were unambiguously positioned in the FAD-binding site of each protomer, based on a strong difference electron density (Figure 6B). The dimer organization is such that the FAD-binding clefts of the two protomers are joined into one large trough on the same face of the CtcP dimer. As expected, FAD was bound to CtcP at a highly conserved site, with most FAD interacting residues identical or similar to those of PltM (Supplementary Figure S1). We previously identified an FAD sensor loop (residues 186–192; Figure 6B) in PltM (residues 172–178). In PltM, this loop changed its conformation depending on the extent of FAD binding [14]. All but two internal residues of this loop in CtcP are identical or similar to those in PltM (Supplementary Figure S1), and this loop is in the same conformation as its PltM counterpart in the state where FAD is fully bound, as expected.

### Crystal structure of CtcQ

**Figure 7:**
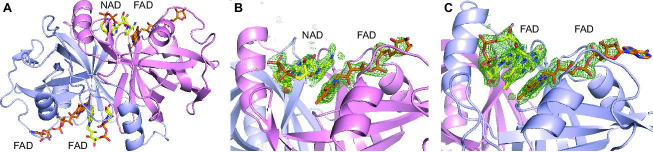
Crystal structure of FAD reductase CtcQ. (**A**) A CtcQ dimer with one protomer (in pink) bound to NAD and FAD and the other (in blue) bound to two FAD molecules. (**B**) A magnified view of the active site of the protomer bound to NAD and FAD. (**C**) A magnified view of the active site of the protomer bound to two FAD molecules. The green mesh in panels **B** and **C** is the omit *F*_o_-*F*_c_ electron density map contoured at 3σ.

The *ctcQ* gene encoding the FAD reductase CtcQ is adjacent to *ctcP* [16], making CtcQ a likely partner of CtcP in a two-component halogenase-reductase system. CtcQ was previously shown to be an efficient NADH-dependent FAD reductase [16]. We cloned, expressed, and purified CtcQ, which was highly active with either FAD or FMN as a substrate (Supplementary Figure S3), in agreement with the previous results. We crystallized and determined the crystal structure of CtcQ in complex with FAD and NAD (Figure 7A). As expected from the sequence homology with other structurally characterized flavin reductases, including SgcE6, TftC, and HpaC, which are ~35% pairwise identical in sequence to CtcQ, CtcQ is a dimeric class I HpaC-like flavin reductase. These proteins contain a large β-sheet that forms the dimeric interface as well as an active site cleft large enough to accommodate both NAD and FAD. Despite modest sequence conservation, including the residues interacting with the NAD and FAD (Supplementary Figure S4), the structure of the proteins in this class is highly conserved. The crystals of the CtcQ-NAD-FAD complex contained four CtcQ dimers in the asymmetric unit. Remarkably, in two of the dimers, both active sites contained two molecules of FAD in each, whereas in two other dimers, one active site contained two molecules of FAD and the other contained one FAD and one NADH molecule (Figure 7B and C). Binding of two FAD molecules in the same active site is not surprising and results from a high concentration of FAD in the crystallization solution and a high affinity of CtcQ for an adenine nucleotide binding to both the co-substrate (NAD) and the substrate (FAD) subsites. Analogous binding of two FAD molecules in the same active site was previously reported for the dimeric corrin reductase CobR (PDB ID: 4IRA [20]). In all cases, the FAD and NAD ligands were well discerned and modeled based on a strong omit *F*_o_-*F*_c_ difference electron density (Figure 7B and C). The bound NADH adopted a U-shaped conformation, as previously observed in other flavin reductase structures [21], with the nicotinamide ring stacked with the isoalloxazine ring of the interacting FAD bound in an extended conformation (Figure 7B). In the active sites occupied by two FAD molecules, the FAD bound in place of NAD was also in a U-shaped conformation, and the two isoalloxazine rings were stacked analogously.

### Weak association of CtcP and CtcQ

**Figure 8:**
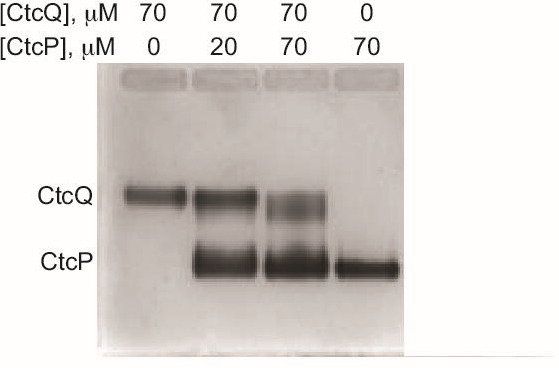
Electrophoretic mobility shift assay demonstrating weak CtcP-CtcQ association on a 1.7% agarose gel. The smearing of the CtcQ and CtcP bands towards one another is indicative of dissociation of the CtcP-CtcQ complex during the electrophoretic migration.

Two-component halogenase-flavin reductase and monooxygenase-flavin reductase complexes have not been tractable as complexes by crystallographic or electron microscopy structural studies due to a low affinity of these complexes, with the *K*_d_ likely in the μM range. Formation of such complexes has been challenging to observe by other techniques [22]. We set out to examine the potential formation of the CtcP-CtcQ complex by an electrophoretic mobility shift assay (EMSA) using a native agarose gel [23] (Figure 8). CtcP and CtcQ migrated as well-defined and distinct bands. Upon titrating CtcP into CtcQ, no new band was observed, indicating the absence of a long-lived, highly stable complex of these two proteins. However, a clear shift and/or smearing of the CtcQ band toward the CtcP band and, conversely, some smearing of the CtcP band towards the CtcQ band were apparent, becoming more prominent with increasing concentration of CtcP. These effects indicated the two proteins formed a complex that was dissociating during electrophoresis, resulting in CtcQ migrating faster and CtcP migrating more slowly in a mixture than individually. We suggest that the handover of FADH_2_ from CtcQ to CtcP for reduction takes place when the CtcP-CtcQ complex is formed.

### Discussion on the structural models of the CtcP-CtcQ complex

**Figure 9:**
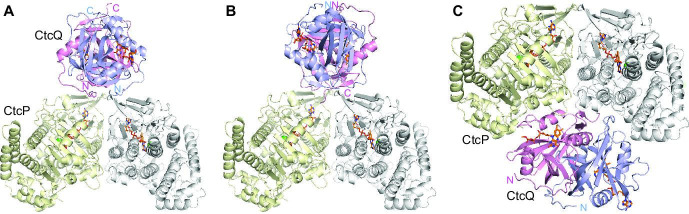
Models of the CtcP-CtcQ complex. (**A**) A model built based on the symmetry and proximity of FAD-binding sites of the two proteins. (**B**) An alternative model built based on the symmetry and proximity of FAD-binding sites of the two proteins. (**C**) The HDOCK model of the CtcP-CtcQ complex. The FAD molecules bound to CtcP and CtcQ are shown as orange sticks. The N- and C-termini of CtcQ are labeled.

Having obtained experimental evidence of CtcP-CtcQ complex formation and the crystal structures of the CtcP and the CtcQ dimers, we constructed three model arrangements of CtcP and CtcQ in their complex (Figure 9). The dimeric organization of both CtcP and CtcQ suggested that the dyad axes of the two proteins might coincide in the complex, to allow for symmetrical shuttling of FAD(H_2_) from one enzyme to the other. Furthermore, in our models, CtcQ is bound to the FAD-binding face of the CtcP in the orientation that minimizes the distance that FAD(H_2_) must travel between the enzymes. Because the active sites in CtcQ are located on opposite faces of the protein, a rotational ambiguity for CtcQ arises, where either the N- or the C-terminal face of CtcQ can contact CtcP while satisfying the above conditions. This ambiguity resulted in two models. In one (Figure 9A), the N-termini of CtcQ are located in the interface with CtcP. In the other (Figure 9B), CtcQ is rotated 180°, so that its C-termini are in the interface with CtcP and the N-termini are on the opposite side. We note that these models are not based on any physical principles, except for avoiding steric clashes. When we used docking software HDOCK [24] to dock the two enzymes, it yielded CtcQ bound to the cleft on the opposite face of the CtcP dimer (Figure 9C) in an asymmetrical orientation and with high surface complementarity of CtcP and CtcQ in the interface. This model has a high docking score (-240) suggestive of likely protein–protein binding. In this model, the FAD-binding sites of the two enzymes are separated by the body of CtcP. It is possible that minimizing the distance between the FAD-binding sites is not necessary, and that the presumably more stable binding in the HDOCK model is more advantageous than the transient binding ensuring the closer approach of the FAD-binding sites. Nevertheless, our EMSA data show weak CtcP-CtcQ binding favoring one of the first two models. If one assumes that the CtcP-CtcQ complex is similar to other two-component halogenase/monooxygenase-FAD reductase complexes, then the model in panel B of Figure 9 is favored, since the CtcP-CtcQ interface in that model involves only structurally conserved core regions of the two enzymes. In contrast, the interactions in panels A and C involve non-conserved N-terminal regions of CtcQ and non-conserved C-terminal regions of CtcP, respectively. The assumption that this complex would need to be similar to other complexes need not be correct, since both enzymes are encoded in the same gene cluster, which could have uniquely evolved. Which, if any, of these models reflects reality remains to be investigated.

## Conclusions

In this study, we determined the crystal structures of halogenase CtcP and FAD reductase CtcQ from the CTC biosynthetic pathway. These enzymes compose a two-component system, and they are weakly associated *in vitro*. Based on the dyad symmetry and the disposition of the FAD molecules bound to these enzymes in the crystal structures, we built a structural model of the CtcP-CtcQ complex. The lack of observed halogenation of TC by CtcP and its small substrate-binding pocket, which cannot accommodate a TC molecule, suggests that this enzyme acts on an earlier pathway intermediate in the cell, and that the subsequent enzymes in the pathway are active on both chlorinated and unchlorinated intermediates. Clearly, the chlorination step in the TC pathway merits further mechanistic investigation.

## Materials and methods

### Molecular cloning, protein expression, and purification

Genomic DNA from *K. aureofaciens* (NRRL #B-5404) was extracted by using a cetyltrimethyl ammonium bromide (CTAB)-based method described in detail previously [25]. Briefly, the cell pellet was resuspended in TE buffer (10 mM Tris pH 8.0, 5 mM EDTA) and incubated with lysozyme at 37°C for 24–26 h. The cell debris was pelleted by centrifugation at 100×g for 1 min. Then the supernatant was used for sodium dodecyl sulfate, proteinase K, and CTAB treatment, and, finally, the genomic DNA was extracted by phenol:chloroform:isoamyl alcohol/25:24:1 and precipitated with isopropanol. After the DNA was pelleted by centrifugation, the pellet was washed with ice-cold ethanol, air-dried, and resuspended in TE buffer. The *ctcP* gene was amplified by a polymerase chain reaction (PCR) from this genomic DNA by using forward and reverse primers (IDT) 5’-ATCAACCACATATGACCGACACAACCGCGGATC-3’ and 5’-ATTCGGATCCTCACGGCTTGACCCGCATCGCCG-3’ (restriction sites are underlined), respectively. The appropriately processed insert was ligated into the pET19b-pps vector [26] between *Nde*I and *BamH*I sites. In this construct, the CtcP protein was expressed with an N-terminal decahistidine tag cleavable with PreScission protease (VWR). The truncated construct of CtcP (residues 1–516, P516G) was generated analogously, by using the reverse primer 5’-ATTCGGATCCTCAGCCGTTGATGTGGTGGGTGTCGGGG-3’.

Full-length and truncated CtcP were expressed in *Escherichia coli* BL21(DE3) cells transformed with the respective pET19b-pps-*ctcP* and pET19b-pps-*ctcP516* vectors. A single colony grown on Luria-Bertani (LB) agar supplemented with 100 μg/ml ampicillin was inoculated in a 5 ml liquid LB medium with 100 μg/ml ampicillin (LB/Amp). The culture was incubated with shaking (200 rpm) at 37°C until the mid-log phase, then inoculated into 2 L of LB/Amp medium. This culture was grown with shaking (200 rpm) at 37°C to an attenuance of 0.4 at 600 nm, transferred and equilibrated at 16°C, induced with 0.4 mM isopropyl β-d-1-thiogalactopyranoside (IPTG) and incubated with shaking (200 rpm) for 16 h at 16°C. The cells were pelleted by centrifugation at 5000×g for 10 min. The cell pellet was resuspended in lysis buffer (40 mM Tris pH 8.0, 1 M NaCl, 10% (*v*/*v*) glycerol). The cells were disrupted by sonication, and the cell debris was pelleted by centrifugation at 40,000×g for 40 min. The proteins were then purified by Ni^2+^-chelating chromatography on a 5 ml HiTrap IMAC FF column (Cytiva), as recommended by the manufacturer. We used a Ni^2+^ column buffer (40 mM Tris pH 8.0, 0.4 M NaCl, 10% (*v*/*v*) glycerol) for column equilibration. After sample loading, the column was washed with 100 ml of the wash buffer (40 mM Tris pH 8.0, 0.4 M NaCl, 10% (*v*/*v*) glycerol, 50 mM imidazole). The proteins were eluted with 10 ml of the elution buffer (40 mM Tris pH 8.0, 0.4 M NaCl, 10% (*v*/*v*) glycerol, 500 mM imidazole). The tag was cleaved with PreScission protease overnight at 4°C, and the digested proteins were further purified on a size-exclusion S-200 column (Cytiva) equilibrated in the gel filtration buffer (40 mM Tris pH 8.0, 100 mM NaCl, and 2 mM β-mercaptoethanol (BME)). The protein-containing fractions were pooled, and the proteins were concentrated using Amicon centrifugal units (EMD Millipore) to 10 mg/ml for crystallization and the full-length CtcP-CtcQ-binding assay.

The *ctcQ* gene was amplified by PCR from the same genomic DNA using forward and reverse primers 5’-ATCAACCACATATGCCCCCCGAACCCCTGTCC-3’ and 5’-ATCCTCGAGGCCCTCGGCCGCCAGGGTC-3’, respectively, and ligated into pET22b between *Nde*I and *Xho*I restriction sites. The resulting protein contained a C-terminal hexahistidine tag. In addition, we generated CtcQ analogously to CtcP, with a removable N-terminal decahistidine tag. To make this construct, PCR was carried out with primers 5’- ATCAACCACATATGCCCCCCGAACCCCTGTCC-3’ and 5’-ATCCTCGAGTCAGCCCTCGGCCGCCAGGGTC-3’ and ligated into pET19b-pps between *Nde*I and *Xho*I restriction sites. Both C- and N-terminally tagged CtcQ proteins were expressed and purified by Ni^2+^-chelating chromatography similarly to CtcP, with the exception that for the C-terminally tagged CtcQ, 20 mM and 200 mM imidazole were used in the wash and the elution buffers, respectively. The C-terminally tagged protein was then dialyzed against dialysis buffer (20 mM Hepes pH 7.5, 10 mM NaCl, 10% (*v*/*v*) glycerol, and 2 mM BME) and concentrated analogously to CtcP for the reductase activity assay. For the N-terminally tagged CtcQ, the tag was removed similarly to CtcP, followed by the S-200 size-exclusion chromatographic step, which was carried out in 20 mM HEPES pH 7.5, 2 mM BME. The protein-containing fractions were pooled, and the protein was concentrated to 12 mg/ml for crystallization and the CtcP-CtcQ-binding assay.

### Crystallization and crystal structure determination

Both CtcP and CtcQ were crystallized by the vapor diffusion method in hanging drops. To obtain crystals of CtcP, we mixed 1 μl of concentrated CtcP and 1 μl of a reservoir solution (100 mM HEPES pH 7.0, 1.6 M ammonium sulfate, 2% (*v*/*v*) PEG 400). These drops were incubated against 1 ml of reservoir solution at 21°C. The crystals were gradually transferred into a cryoprotectant solution (0.1 M HEPES pH 7.0, 1.6 M ammonium sulfate, 2% (*v*/*v*) PEG 400, 20% (*v*/*v*) glycerol), incubated for 30 min and frozen in liquid nitrogen by quick immersion. To obtain the crystals of the CtcP-FAD complex, we set the drops by mixing 1 μl of CtcP with 1 μl of a different reservoir solution (0.1 M HEPES pH 7.5, 26% (*w*/*v*) PEG 4000, 0.2 M Li_2_SO_4_) and incubated against this reservoir solution at 21°C. The crystals were then gradually transferred into a cryoprotectant solution containing FAD (0.1 M HEPES pH 7.5, 34% (*w*/*v*) PEG 4000, 0.2 M Li_2_SO_4_, 20 mM NaCl, 1 mM FAD, 15% (*v*/*v*) glycerol), incubated for 16 h and then frozen analogously to the crystals of apo-CtcP. For crystallization of CtcQ in complexes with NAD and FAD, the concentrated protein was incubated with 5 mM NAD and 5 mM FAD on ice for 10 min. Drops contained 1 μl of this mixture and 1 μl of another reservoir solution (0.1 M Tris pH 7.5, 0.6 M KH_2_PO_4_, 0.6 M NaH_2_PO_4_). The drops were incubated against 1 ml of this reservoir solution at 21°C. The solution in the drop with the crystals was then gradually changed to a cryoprotectant solution that had the same composition as the reservoir solution and additionally contained 25% (*v*/*v*) glycerol, 1 mM NAD, and 1 mM FAD. The crystals were incubated in this solution for 30 min and then rapidly immersed into liquid nitrogen.

The X-ray diffraction data were collected at 100 K at beamline 22-ID of the Advanced Photon Source at the Argonne National Laboratory (Argonne, IL). The data were indexed, integrated, and scaled with HKL-2000 [27]. The structure of CtcP-PEG was obtained by molecular replacement with PHASER [28], where a crystal structure of phenolic halogenase PltM (PDB accession code: 6BZQ) was used as the search model. The structure was then iteratively built and refined with Coot [29] and REFMAC [30], respectively. The crystal structure of the CtcP-FAD complex was determined by molecular replacement using PHASER and the crystal structure of CtcP-PEG as a search model. FAD was built into strong *F*_o_-*F*_c_ electron density, followed by analogous structure building and refinement cycles. The crystal structure of the CtcQ-FAD-NAD complexes was determined by molecular replacement using PHASER and a crystal structure of a putative oxidoreductase from *Rickettsia felis* (PDB accession code: 4L82) as a search model. FAD and NAD were built into strong *F*_o_-*F*_c_ electron density, and then this structure was built and refined analogously to the above structures. The three crystal structures were deposited into the Protein Data Bank (PDB). The PDB accession codes and the crystallographic statistics are given in Supplementary Table S1. The solvent-accessible surface area calculations were performed with Surface Racer [31].

### Halogenation activity assays

The halogenation reactions (100 μl) were carried out in the halogenation buffer (40 mM Tris pH 7.4, 100 mM NaCl) at 25°C for 2 h. The full reaction contained 0.1 mM FAD, 1 mM NADH, 5–100 μM CtcP (full-length or the truncation mutant 1–516), 5 μM CtcQ, 0.2 mM TC, 20 mM glucose, and 0.5 μM glucose dehydrogenase from *Bacillus amyloliquefaciens* SB5 (prepared as described previously [14]). Control reactions were set up by omitting one or more of the above species, as specified. As HPLC standards, we used 30 μM TC and 30 μM CTC. The reactions were quenched, and the proteins were precipitated with 0.9 ml of methanol and pelleted by centrifugation at 10,000×g for 10 min. The supernatant was dried in a SpeedVac for 1.5 h without heating, and the dry material was resuspended in 80 μl of milli-Q H_2_O. After centrifugation for 1 min at 10,000×g, 10 μl of supernatant was injected onto HPLC. The HPLC protocol was the same as that published previously [14] using a Vydac Denali C18 column (250 mm × 4.6 mm), except as eluent A we used 0.1% TCA in milli-Q H_2_O. Absorbance at 257 nm was monitored.

### The CtcP-CtcQ binding assay

CtcQ (70 μM) was mixed with CtcP at various concentrations (as specified) in the binding buffer (20 mM HEPES pH 7.5). The binding mixture was incubated on ice overnight and then loaded onto 1.7% (*w*/*v*) agarose gel and run at 4°C in the running buffer (25 mM Tris, 192 mM glycine). The gel was stained by Coomassie blue.

### The reductase activity assay with CtcQ

The reductase activity assay was carried out in 20 mM HEPES (pH 7.5) with 1 mM NADH, 0.1 mM FAD or 0.1 mM FMN, and 1.7 μM CtcQ or no protein and 0.1 mM FAD as a control. The reactions (100 μl) were set up in 96-well plates. The assay was initiated by adding CtcQ, after which the change in absorbance at 340 nm over time was monitored using a SpectraMax M5 (Molecular Devices) microplate reader. The assay was carried out in triplicate.

## Supplementary material

Online supplementary tables

## Data Availability

The crystal structure coordinates and structure factor amplitudes are available at the Protein Data Bank with the following accession codes: CtcP: 7V0D,[32] 7V0B;[33] CtcQ: PDB 8CT0. Other data are available upon request.

## Abbreviations

CTC: chlortetracycline
PCR: polymerase chain reaction
PEG: polyethylene glycol
TC: tetracycline
