# Supplementary material for: Crystal structures and low-affinity complex formation of halogenase CtcP and FAD reductase CtcQ from the chlortetracycline biosynthetic pathway
Source: Biosci Rep. 2025 Jul 4;45(7):401–14. doi: 10.1042/BSR20253185 (PMC12400496; doi:10.1042/BSR20253185)
Supplement: Online supplementary tables [file bsr-45-07-BSR20253185-s001.pdf]

SUPPLEMENTARY INFORMATION

Crystal structures and complex formation of halogenase CtcP and FAD reductase CtcQ from the chlortetracycline biosynthetic pathway

Caixia Hou,[a] Sylvie Garneau-Tsodikova,[a] and Oleg V. Tsodikov[a,*]

[a] Department of Pharmaceutical Sciences, College of Pharmacy, University of Kentucky, 789 South Limestone Street, Lexington, KY, 40536-0596, USA.

* Correspondence should be addressed to oleg.tsodikov@uky.edu

Table S1. Crystallographic statistics for the crystal structures of CtcP in complex with PEG, CtcP in complex with FAD, and CtcQ in complex with FAD and NAD.

	CtcP-PEG	CtcP-FAD	CtcQ-FAD-NAD
PDB accession code	7V0D	7V0B	8CT0
Data collection			
Resolution (Å)	50.0-2.6 (2.64-2.60)[a]	50.00-2.15 (2.19-2.15)	50.00-2.45 (2.49-2.45)
$I/\sigma I$	13 (2.0)	15 (1.93)	9 (2.0)
Completeness (%)	98.8 (99.9)	96.9 (99.1)	96.4 (95.4)
Redundancy	8.6 (8.9)	3.5 (3.4)	3.4 (3.2)
R_{merge}	0.15 (0.89)	0.14 (0.80)	0.138 (0.568)
$CC_{1/2}$	0.989 (0.895)	0.982 (0.685)	0.978 (0.696)
Number of unique reflections	58,486	121,382	49,072
Structure refinement			
Resolution (Å)	35.0-2.6	35.00-2.15	34.97-2.45
Space group	$P2_12_12_1$	$P2_1$	$P2_1$
Protomers per asymmetric unit	2 (1 dimer)	4 (2 dimers)	8 (4 dimers)
Unit cell dimensions:			
a, b, c (Å)	98.6, 106.0, 178.9	62.8, 103.6, 179.2	56.7, 123.6, 102.7
α, β, γ (°)	90, 90, 90	90, 95.4, 90	90, 99.1, 90
N_{atoms}	8,905	18,178	11,093
R_{overall} (%)	18.8	19.0	19.7
R_{free} (%)	21.9	23.3	25.6
r.m.s.d.[b] from ideal			
bond lengths (Å)	0.003	0.004	0.004
bond angles (°)	1.195	1.234	1.349
Ramachandrand plot statistics[c]			
%residues in regions			
favored	96	98	96
allowed	4	2	4
outliers	0 (0 residues)	0 (0 residues)	0 (0 residues)

[a] Numbers in parentheses indicate the values in the highest resolution. shell.

[b] r.m.s.d. stands for root-mean-square deviation.

[c] Ramachandran plot statistics are calculated using MolProbity[1], version 4.02b-467.

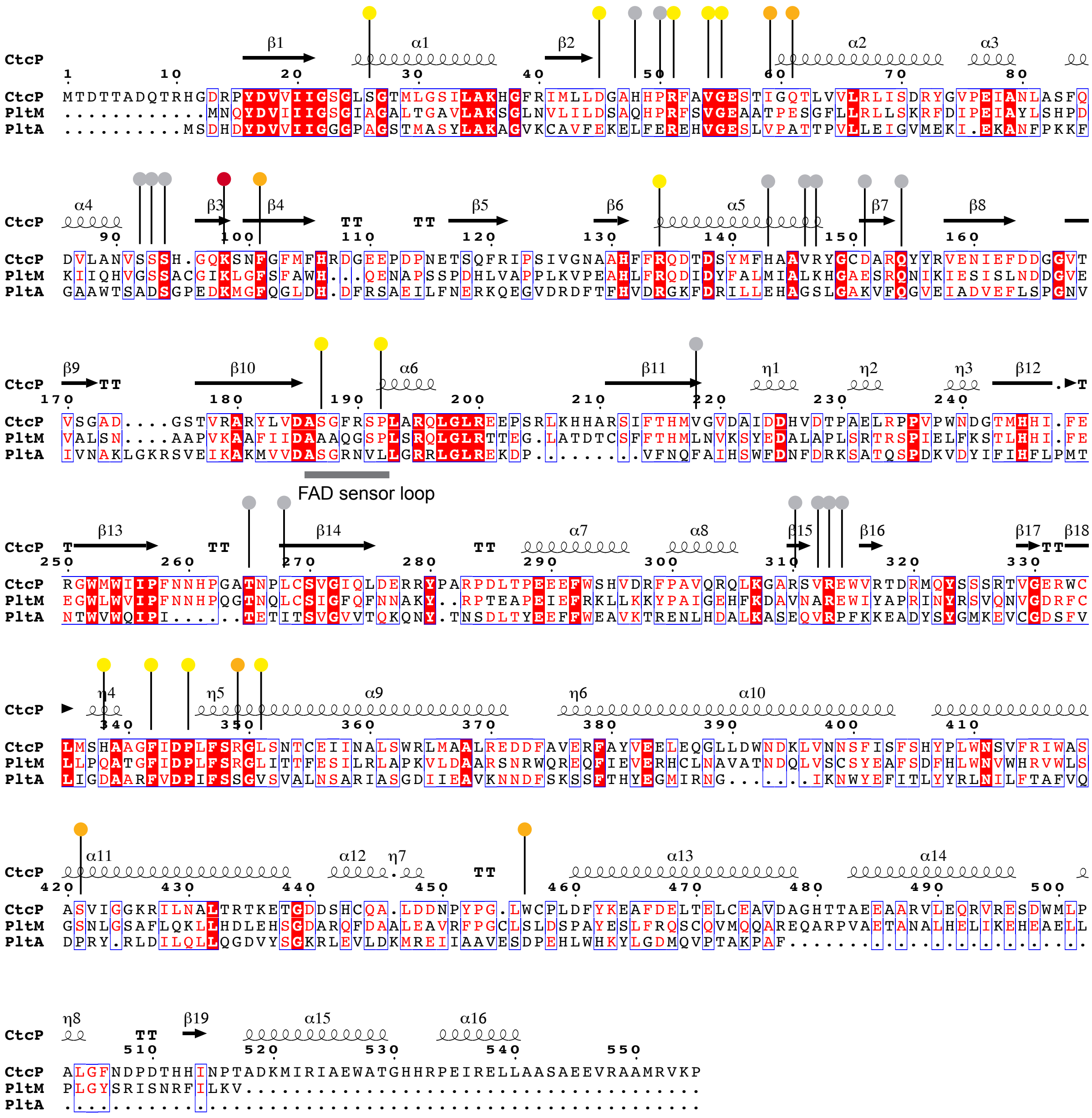

Figure S1. A sequence alignment of CtcP, PltM, and PltA. The residues in the dimerization interface are shown by grey lollipops, those lining the substrate binding pocket by orange lollipops, and those interacting with FAD by yellow lollipops. The catalytic Lys98 is shown by a red lollipop. The alignment was generated by Multalin[2].

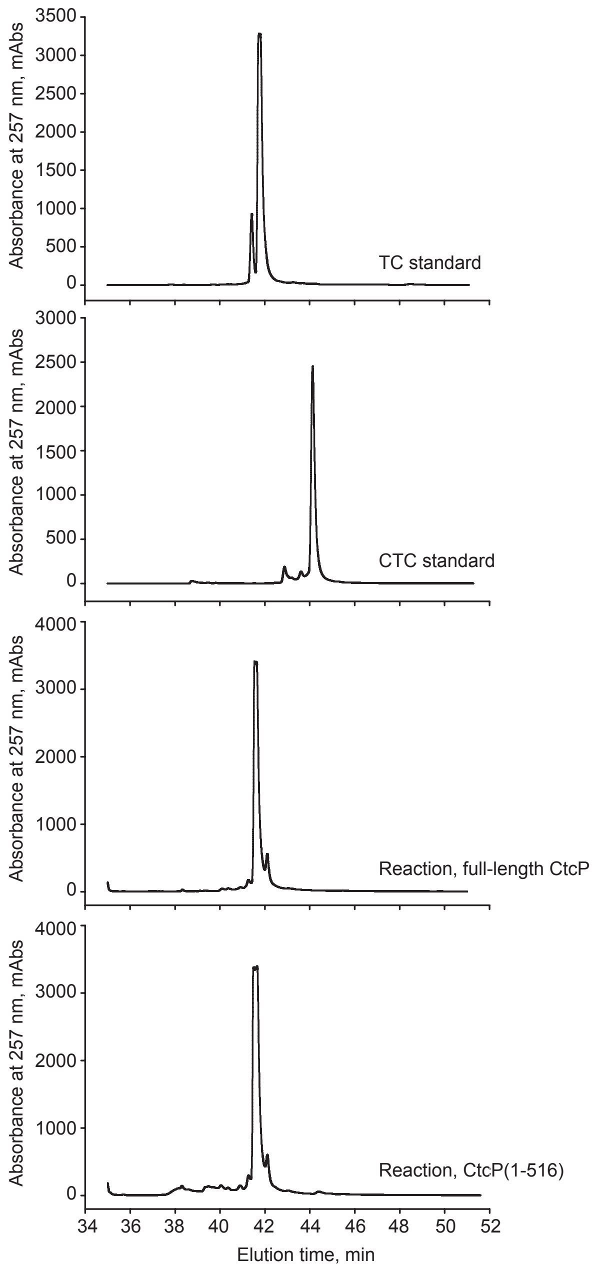

Figure S2. HPLC traces of TC, CTC, and chlorination reactions with full-length CtcP and CtcP(1-516).

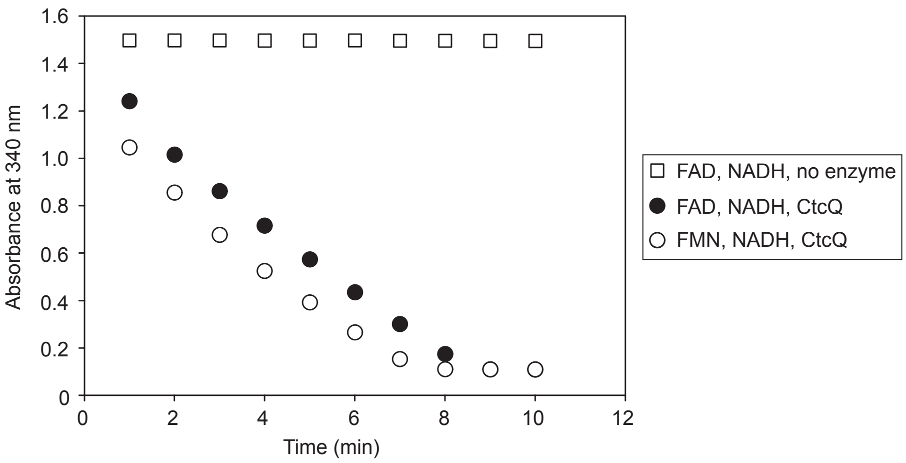

Figure S3. The reductase activity assay with CtcQ. The decrease in absorbance at 340 nm indicates FAD/FMN-dependent oxidation of NADH catalyzed by CtcQ. The symbols are averages of triplicate experiments; the size of the symbols is comparable to the standard deviation of the measurements.

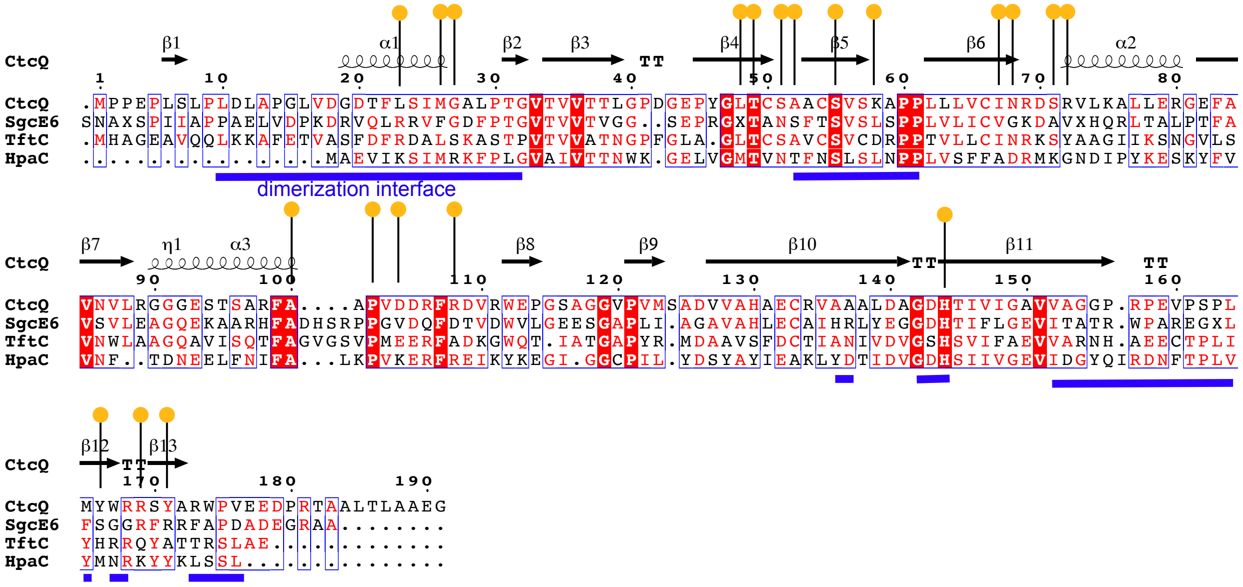

Figure S4. A multiple sequence alignment of CtcQ and representative previously characterized flavin reductases. The residues in the dimerization interface are shown by the blue lines under the alignment. The residues lining the substrate/co-substrate binding pocket are shown by orange lollipops. The alignment was generated by Multalin[2].

REFERENCES

1 Williams, C. J., Headd, J. J., Moriarty, N. W., Prisant, M. G., Videau, L. L., Deis, L. N. et al. (2018) MolProbity: More and better reference data for improved all-atom structure validation. Protein Sci. **27**, 293-315. DOI: 10.1002/pro.3330

2 Corpet, F. (1988) Multiple sequence alignment with hierarchical clustering. Nucleic Acids Res. **16**, 10881-10890. DOI: 10.1093/nar/16.22.10881
